# Posttreatment Surveillance in Patients with Prolonged Disease-Free Survival After Resection of Colorectal Liver Metastasis

**DOI:** 10.1245/s10434-016-5388-8

**Published:** 2016-07-08

**Authors:** Boris Galjart, Eric P. van der Stok, Joost Rothbarth, Dirk J. Grünhagen, Cornelis Verhoef

**Affiliations:** Department of Surgical Oncology, Erasmus MC Cancer Institute, Rotterdam, The Netherlands

## Abstract

**Introduction:**

Posttreatment surveillance protocols most often endure for 5 years after resection of colorectal liver metastasis (CRLM). Most recurrences happen within 3 years after surgical removal of the tumour. This study analysed the need of surveillance for patients with at least 3 years of disease-free survival after potentially curative resection of CRLM.

**Methods:**

A single-centre, retrospective analysis of all consecutive patients who underwent treatment for CRLM with curative intent between 2000 and 2011.

**Results:**

In total, 152 of 545 patients (28 %) remained disease-free for 3 years after successful resection of the CRLM. The estimated recurrence rate after 10 years of follow-up in this group of 152 patients was 27 %. More than half of these patients (55 %) could be treated with curative intent for their recurrences. Multivariable analysis revealed that the nodal status of the primary tumour is of significant prognostic value for developing recurrences after 3 years of disease-free survival. A disease-free interval of less than 12 months between resection of primary tumour and detection of CRLM shows a trend towards significance. Both factors were used to create a risk score, showing that patients with a low-risk profile (node-negative status and a disease-free interval <12 months) have an estimated recurrence rate of 5 % and might not benefit from intensive surveillance beyond 3 years of follow-up without a recurrence.

**Conclusions:**

The currently developed risk score shows that follow-up can be stopped in a specific subgroup 3 years after treatment for their CRLM with curative intent.

**Electronic supplementary material:**

The online version of this article (doi:10.1245/s10434-016-5388-8) contains supplementary material, which is available to authorized users.

Liver metastases are common in patients with colorectal cancer (CRC), developing in approximately half of patients with colorectal tumours.^1^,^2^ Surgical treatment of colorectal liver metastasis (CRLM) results in 5-year overall survival (OS) of 40–60 %.^3^,^4^ Although the treatment of CRLM has improved, disease recurrence is seen in almost 70 % of the patients. Most often recurrences develop during the first 3 years after surgery.^5^–^7^ Both hepatic and pulmonary recurrences can be treated with local therapy repeatedly, thereby still offering the potential of cure.^8^–^13^ The opportunity to control recurrent disease as a curable condition increased interest in the surveillance of patients after hepatectomy. No consensus on the optimal follow-up protocol for curatively treated patients with stage IV CRC has been reached however.

Patients treated with curative intent for CRLM enter a surveillance scheme, enduring for 5 years in most centres. Research on the surveillance and prognosis of patients with CRLM mainly focuses on the first 3 years after surgery, because most recurrences happen during this period. Literature is scarce on the follow-up of patients with a disease-free survival (DFS) of 3 years and more.^14^ The current study was designed to analyse the need for surveillance in these patients by determining the recurrence pattern, treatment for recurrences, and oncological outcome. This study assessed the possibilities for a risk-based surveillance protocol in this highly selected but growing group of patients.

## Patients and Methods

Patient data were extracted from a prospectively maintained database in Erasmus MC Cancer Institute. The database consists of perioperative and clinicopathological characteristics of primary CRC, CRLM, and recurrent metastatic disease. In this retrospective analysis, patients who received surgical or ablative therapy for CRLM between January 2000 and November 2011 were included. In this group, all patients with a DFS of more than 3 years were identified. In case of relapsing disease after liver surgery, data on recurrence location, diagnosis, and treatment were collected.

### Follow-Up of Patients with CRLM

Surveillance consisted of physical examination, thoracoabdominal computed tomography (CT) and regular serum carcinoembryonic antigen (CEA) level measurements. Patient surveillance was performed for up to 5 years after treatment of CRLM. During this period, serum CEA measurements and radiological imaging were performed every 3–6 months during the first 3 years after surgery and yearly thereafter.

### Recurrent Disease

In the present study, recurrences detected within 3 years of CRLM treatment with curative intent were categorized as early recurrences. All recurrences detected after 3 years were considered to be late recurrences. CEA blood levels >5.00 µg/L were considered elevated. In case of normal CEA levels, the absolute difference between baseline postoperative CEA levels and CEA levels at time of recurrence was calculated.

Treatment of recurrent disease was assessed in a multidisciplinary tumour board for all patients. Because long-term local control of metastatic CRC is achieved using surgery, radiofrequency ablation (RFA), or stereotactic radiotherapy (SRx), all of these modalities were considered to be potentially curative treatments for recurrent disease.^15^,^16^

### Disease-Free and Overall Survival

Disease-free survival was calculated as the time in months between the resection of CRLM and the diagnosis of recurrent disease (either by radiology, physical examination, or endoscopy). When an elevated CEA level was the first sign of possible recurrence, this was followed by confirmative imaging or biopsies. The dates of the latter were used for survival calculations.

Overall survival was the time between treatment of CRLM and the date of death or last follow-up. For both patients with a DFS of 3 and 5 years, conditional OS and DFS curves were created, using 36 and 60 months as the starting points (*t*_0_). To compare oncological outcome after potentially curative treatment for early and late recurrences, the survival estimate DFS2 (from start treatment of recurrence until re-recurrence) was calculated.

### Statistical Analysis

The categorical data are presented as absolute numbers and percentages. Continuous variables were displayed as means (and standard deviations) or medians (and interquartile ranges). Different proportions between groups were tested using the Chi squared test. Univariable and multivariable regression models were created to identify factors related to late disease recurrence, for which hazard ratios (HR) and 95 % confidence intervals (CI) were calculated. Prognostic factors were used to create a risk score. The score was internally validated for discrimination (concordance index) and calibration (calibration plot), using bootstrap resampling. The Kaplan–Meier method was used to estimate (conditional) survival. All (conditional) survival estimates were compared using the log-rank test. A *p* value <0.05 was considered significant. All analyses were performed using SPSS version 21.0 (SPSS Inc., Chicago, IL) and R version 3.2.5 (http://www.r-project.org).

## Results

**Fig. 1:**
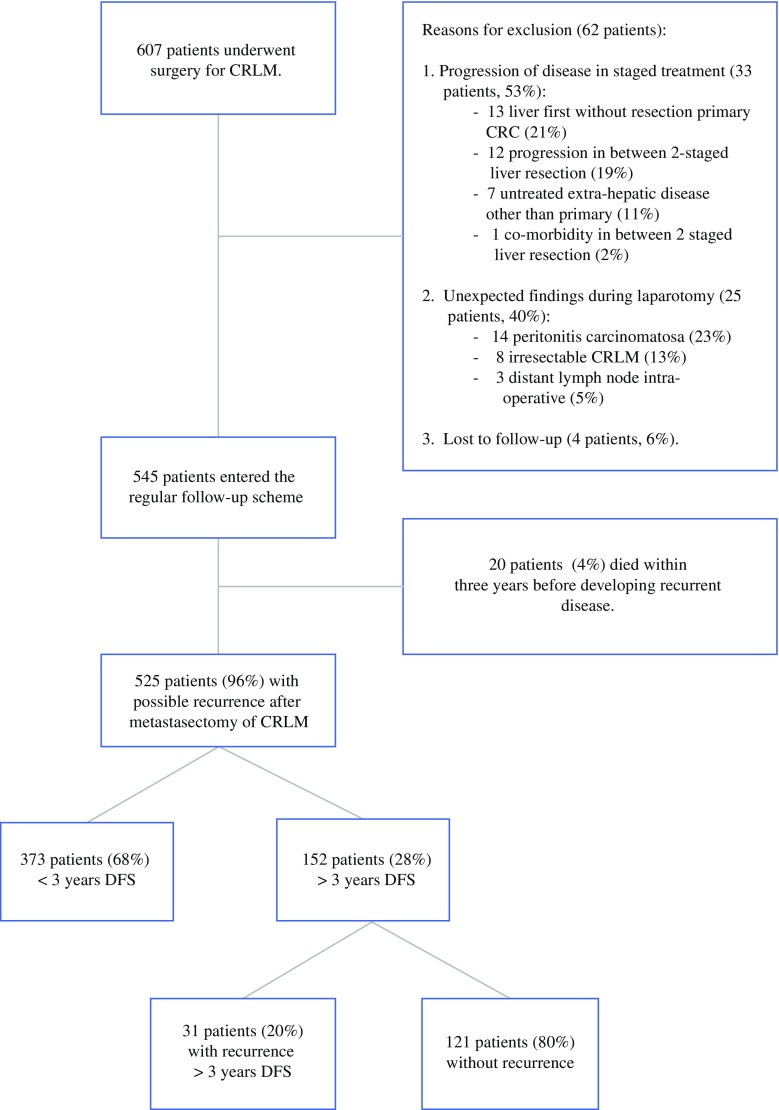
Flowchart of the study

Of the 607 patients with a minimal potential follow-up of 3 years and potentially curative treatment for CRLM, 545 consecutive patients (90 %) were eligible for analysis in this study. Exclusion criteria are presented in Fig. 1. A total of 152 patients were disease-free after 3 years of follow-up (28 %), of which 31 patients (20 %) developed recurrences beyond 3 years. Median follow-up time (*t*_0_ = 36 months after first hepatectomy) was 40 months (interquartile range: 18–63 months) in this group. Twenty-four patients (16 %) died during the follow-up period. In patients with 3 years of DFS, the Kaplan–Meier analysis showed an estimated recurrence rate of 27 % in the following 7 years of follow-up.

**Fig. 2:**
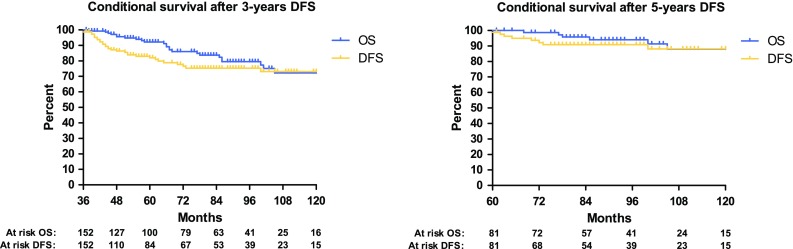
Conditional DFS and OS for patients with 3 and 5-years DFS

Eighty-one patients were disease-free for more than 5 years (15 %). Median follow-up time in this group of patients (*t*_0_ = 60 months after first hepatectomy) was 31 months (interquartile range: 20–52 months). Seven recurrences (9 %) and six deaths (7 %) were observed, and the estimated (Kaplan–Meier) probabilities of recurrence and mortality in the following 5 years were 11 and 12 % respectively. Conditional OS and DFS curves are presented in Fig. 2 for patients with 3 and 5 years of DFS. In total, 393 patients (72 %) had a DFS of less than 3 years. When comparing the recurrence pattern of early (<3 years DFS) and late recurrences (>3 years DFS), no significant differences in tumour location were seen (Table 1).

**Table 1:** Recurrence pattern, surveillance and treatment results

	Recurrence <3 years (*N* = 373)	Recurrence >3 years (*N* = 31)	*p*-value
Location recurrence			
Intrahepatic only	144 (39 %)	9 (29 %)	0.291
Extrahepatic location recurrences	229 (61 %)	22 (71 %)	0.904
Pulmonary recurrence	84 (23 %)	11 (36 %)	
Local recurrence	15 (4 %)	1 (3 %)	
Distant lymph nodes	21 (6 %)	1 (3 %)	
Hepatic and pulmonary	35 (9 %)	1 (3 %)	
Hepatic and other	28 (8 %)	4 (13 %)	
Pulmonary and other	15 (4 %)	2 (7 %)	
Multi-organ metastasis (≥3)	10 (3 %)	1 (3 %)	
Other locations	21 (6 %)	1 (3 %)	
Surveillance			
Median CEA (IQR) μg/L	7.0 (2.9–20.0)	7.1 (3.9–12.7)	0.849
Elevated CEA (>5.0 μg/L)	204 (55 %)	22 (71 %)	0.087
Non elevated CEA (<5.0 μg/L)	152 (40 %)	8 (26 %)	
Missing CEA values	17 (5 %)	1 (3 %)	
Perc. increase (when normal CEA)	152 (40 %)	8 (26 %)	0.255
>25 % compared to baseline	49 (29 %)	4 (50 %)	
1–25 % compared to baseline	25 (15 %)	2 (25 %)	
Decreased compared to baseline	26 (16 %)	2 (25 %)	
Not calculated	52 (34 %)	0 (0 %)	
Treatment			
Curative	168 (45 %)	17 (55 %)	0.293
Non-curative	205 (55 %)	14 (45 %)	

After evaluation of the late recurrences, 17 patients (55 %) could be treated with curative treatment modalities compared with 168 (45 %) of the early recurrences (*p* = 0.293). For patients with curatively treated early recurrences, re-recurrence occurred earlier than in patients with curatively treated late recurrences. Median time to relapse (DFS2) was 28 months (75th percentile at 12 months, 25th not reached) in patients with late recurrences and 8 months (interquartile range: 4–30 months) in patients with early recurrences (*p* = 0.041). Table 1 displays treatment and surveillance results of early and late recurrences.

To define patients who potentially could be excluded from follow-up, the Chi squared test and univariable Cox regression analysis were performed. Factors associated with developing late disease recurrences were the nodal status of the primary tumour, the absence of neoadjuvant chemotherapy for CRLM, and the disease-free interval (DFI) between resection of the primary CRC and the detection of CRLM. The Clinical Risk Score (CRS) described by Fong et al. showed no additional value in assessing the probability of developing late recurrence.^17^

After multivariable analysis, the nodal status remained a statistically significant prognostic factor for late disease recurrence after an initial DFS of 3 years. A DFI of more than 12 months between resection primary and development CRLM) shows a trend towards significance (Table 2).

**Table 2:** Baseline characteristics of patients with 3 years of DFS and the results of univariable and multivariable analysis

Variables	Total (*N* = 152)	Recurrence >3 years (*N* = 31)	Chi-squared *p*-value	Univariable [HR (95 % CI) *p*-value]	Multivariable [HR (95 % CI) *p*-value]
Gender					
Male	94	19 (20.2 %)	0.943	0.942 (0.456–1.943)	
Female	58	12 (20.7 %)		0.871	
Age					
Median (range)	64 (30–86)	66 (30–86)	0.326	1.030 (0.994–1.067)	
Mean ± SD	63.3 ± 11.1	65.9 ± 13.2		0.106	
Primary CRC					
Location					
Colon	93	19 (20.4 %)	0.989	0.978 (0.475–2.015)	
Rectum	59	12 (20.3 %)		0.952	
T-stage					
T3-4	37	3 (8.1 %)	0.086	3.250 (0.989–10.682)	
Tl-2	114	28 (24.6 %)		0.052	
Node status					
Positive	72	20 (27.8 %)	0.035	2.316 (1.109–4.837)	2.279 (1.090–4.764)
Negative	79	11 (13.9 %)		0.025	0.029
Adjuvant CTx					
Yes	31	9 (29.0 %)	0.181	1.890 (0.868–4.116)	
No	121	22 (18.2 %)		0.109	
CRLM					
DFI <12 months					
Yes	93	12 (12.9 %)	0.004	0.372 (0.180–0.766)	0.471 (0.215–1.029)
No	59	19 (32.2 %)		0.007	0.059
Number of CRLM					
>1	75	14 (18.7 %)	0.602	1.002 (4.94–2.033)	
1	77	17 (22.1 %)		0.996	
Size of tumours					
≥5.00 cm	26	7 (26.9 %)	0.386	1.382 (0.595–3.210)	
4.99 cm	124	24 (19.4 %)		0.451	
CEA preoperative					
≥200 μg/L	8	1 (12.5 %)	0.305	0.045 (0.00–46.585)	
≤199 μg/L	120	22 (18.3 %)		0.381	
Bilobar metastases					
Yes	43	9 (20.9 %)	0.918	1.218 (0.560–2.647)	
No	109	22 (20.2 %)		0.691	
Neoadjuvant CTx					
Yes	70	8 (11.4 %)	0.011	0.411 (0.184–0.920)	0.577 (0.241–1.380)
No	82	23 (28.0 %)		0.03	0.216
Margin <1 mm					
Yes	22	4 (18.2 %)	0.743	0.985 (0.344–2.815)	
No	127	27 (21.3 %)		0.977	
EHD					
Yes	3	0 (0.0 %)	0.376	0.048 (0.00–8158.217)	
No	149	31 (20.8 %)		0.621	
Clinical risk score					
HR (3–5)	39	7 (17.9 %)	0.550	0.809 (0.347–1.886)	
LR (0–2)	102	23 (22.5 %)		0.624	

Missing values were observed for T-stage (1), nodal status (1), tumour size (2), preoperative CEA (24), margin status (3 patients with RFA only) and the Clinical Risk Score (11)

*CTx* chemotherapy, *EHD* extra-hepatic disease, *CEA* carcinoembryonic antigen, *LR* low-risk, *HR* high-risk

Risk categories for late recurrences were created, in which patients with node-negative primary tumours and a DFI of less than 12 months (*n* = 50, 33 %) were considered at low-risk. All other patients (with either a N+ status, a DFI of more than 12 months, or a combination of both characteristics) were considered at high-risk of late recurrence (*n* = 101, 66 %). In one patient, no risk score could be determined. In the low-risk group, two patients (4 %) developed recurrence during the following 2 years of surveillance (after the initial 3 disease-free years) compared with 22 patients (22 %) in the high-risk group. The estimated 10-year recurrence rate in the low-risk group was 5 % and was 25 % in the high-risk group (*p* = 0.005). The sensitivity of this risk score for prediction of late recurrences during the last 2 years of follow-up was 92 %. The estimated difference in recurrence rate between the “high-risk” group and the complete group of patients with 3 years of DFS is 2 %. This means that 50 patients with a DFS of 3 years need to remain in follow-up for another 2 years to detect 1 “low-risk” patient with late recurrent disease.

**Fig. 3:**
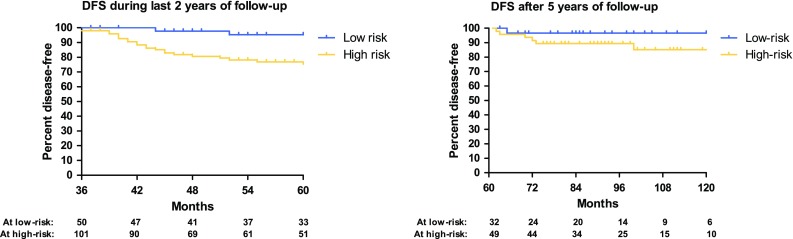
Risk stratification for late recurrences. The *graph* on the *left* illustrates the DFS during the last 2 years of follow-up (from 36 to 60 months after hepatectomy). The *graph* on the *right* illustrates the DFS after more than 60 months after hepatectomy

After 5 years of DFS, one recurrence (3 %) was observed in the low-risk group (*n* = 32) compared with six recurrences (12 %) in the high-risk group (*n* = 49). The estimated 10-year recurrence rate in the following 5 years (after 5 years of DFS) was 3 % in the low-risk group versus 15 % in the high-risk group (*p* = 0.207). Kaplan–Meier curves after 3 and 5 years of DFS are presented in Fig. 3.

The created risk model had a moderate capacity to predict late disease recurrence (bootstrap corrected concordance index: 0.71) and acceptable calibration (see Supplementary Material).

## Discussion

The current study demonstrates that a considerable proportion of patients with a DFS of more than 3 years develop recurrences, with an estimated 10-year recurrence rate of 27 %. Patients with late recurrences received potentially curative treatment as often as patients with early recurrences did. This may justify surveillance in patients with CRLM, even after a DFS of 3 years.

To date, no prospective trials have investigated the efficacy of long-term follow-up of patients with CRLM, nor curatively treated stage IV CRC in general. It is still unclear to what extend surveillance is useful. The primary target of this study was to objectify the necessity of surveillance in patients without evidence of disease 3 years after the first liver metastasectomy. Several groups have shown that repeat resections of recurrences offer survival benefit and although the efficacy of RFA and SRx has been studied less intensively, results indicate that long-term disease control can be reached using these treatments.^15^,^16^,^18^–^22^ Because more than half of the patients with late recurrences were treated with either one or a combination of local treatments, surveillance seems legitimate in this particular group of patients.

Follow-up in the centre of the current study is performed during 5 years for all patients after resection of CRLM, which is advised in the ASCRS and NCCN guidelines.^23^,^24^ Preferably cancer surveillance should only be performed in those patients benefiting from it. In order to decide in which patients follow-up is desirable, accurate prediction of outcome after metastasectomy is needed. Many efforts to determine prognosis of patients with CRLM have been made, of which the CRS is mostly practised.^4^,^17^,^25^ Less evidence is available to predict the likelihood of late disease recurrence, which is demonstrated by the fact that patients with initially poor prognostic factors can still be cured from CRLM.^28^ A study by Tan et al. showed that the currently used risk scores for CRLM have little predictive value in 3-year survivors of CRLM with regards to the disease-specific survival and therefore are not suitable to decide whether long-term follow-up is appropriate.^29^ In the current study, the nodal status of the primary CRC showed to be the only significant prognostic factor with respect to developing late disease recurrence. The DFI was nonsignificant in multivariable analysis but showed a trend towards significance. The interval between resection of the primary tumour and occurrence of CRLM has been used in most CRS, as a DFI of less than a year increases the chance of developing recurrent disease shortly after hepatectomy.^4^,^17^,^25^–^27^ The results in this study indicate an opposite effect in patients with 3 years of DFS, as patients with a short interval (<12 months) between the primary CRC and the occurrence of CRLM had a favourable outcome in this particular group of patients. Although counterintuitive, this finding might not be illogical. In many studies, a short DFI is described as a risk factor for early recurrence and a surrogate for aggressive tumour behaviour, inherently.^17^ Moreover, this means that if patients with a short DFI develop recurrences, it is more likely that these will occur in the period shortly after partial hepatectomy rather than after a period of 3 years. This study showed that in the period thereafter, patients with a short DFI had a lower risk of developing recurrence, because it is unlikely that patients with initially aggressive tumour behaviour will develop recurrences after remaining disease-free for such a significant period of time. Consequently, patients with a prolonged DFI have a decreased risk in the period shortly after surgery but remain at higher risk of recurrence for an expanded period. Considering the more latent tumour behaviour in patients with a prolonged DFI, this might not be implausible. Although patient selection, rather than tumour biology, also could explain the observed effect, this finding might be of interest when considering long-term surveillance in patients with CRLM and therefore should be validated in an external cohort of patients.

To identify patients who could potentially be discharged from (intensive) surveillance, a stratification system was created using both the DFI and nodal status as variables. Patients with optimal prognostic factors (pN0 status and a DFI <12 months) were considered to be at low-risk, resulting in an estimated recurrence probability of 5 %. The results display that this is lower than the estimated 12 % recurrence probability after 5 years of DFS, when it is generally accepted to discharge patients from follow-up. The risk score showed moderately good discrimination and acceptable calibration. Although this scoring system needs external validation and potentially could be extended with other variables, this study indicates that there may be patients with a low-risk profile who do not benefit from a surveillance protocol consisting of 5 years and can either be discharged from follow-up after 3 years or undergo less intensive surveillance by the general practitioner.

During the past decade, several research groups have retrospectively evaluated the different aspects of follow-up after metastasectomy to define an optimal surveillance protocol.^30^–^37^ Jones et al. highlighted the lack of evidence surrounding surveillance of patients with CRLM after reviewing all available literature on early intensive follow-up after metastasectomy and therefore remained inconclusive on how to perform optimal follow-up.^14^ In a review by Metcalfe et al. 5 years of follow-up was proposed.^38^ As shown in this and other studies, patients with a DFS of 5 years still have a probability of approximately 10 % to develop recurrences after being discharged from surveillance. Recent literature stated that cure after resection of CRLM might only be achieved after 10-year survival.^28^,^39^ This suggests that an extended follow-up protocol of more than 5 years could be worthwhile for some patients, again addressing the need for tailor-made follow-up schedules.

The current study has several limitations and its conclusions should be interpreted with care. As a result of the retrospective nature of this study, the obtained results might be biased. Due to the limited number of events after 3 years of DFS, only three factors could be evaluated in the multivariable analysis. It is likely that other factors are influential, although nonsignificant in this particular univariable analysis. Also, the identified risk score has not been externally validated, which impairs generalizability.

Nevertheless, this study provides valuable insights regarding the follow-up of patients with 3 years of DFS after surgery for CRLM. The data suggest that follow-up in patients surviving 3 years without evidence of disease is useful and necessary in most patients. Patients with the currently developed low-risk profile might not benefit from the additional 2 years of surveillance, and patients with a high-risk profile should be followed beyond 5 years, which emphasizes the importance of a tailor-made, long-term, follow-up protocol after treatment of CRLM with curative intent.

## Electronic supplementary material

Below is the link to the electronic supplementary material.

Supplementary material 1 (TIFF 1813 kb)
