# Supplementary figures and images for: Posttreatment Surveillance in Patients with Prolonged Disease-Free Survival After Resection of Colorectal Liver Metastasis
Source: Ann Surg Oncol. 2016 Jul 8;23(12):3999–4007. doi: 10.1245/s10434-016-5388-8 (PMC5047936; doi:10.1245/s10434-016-5388-8)

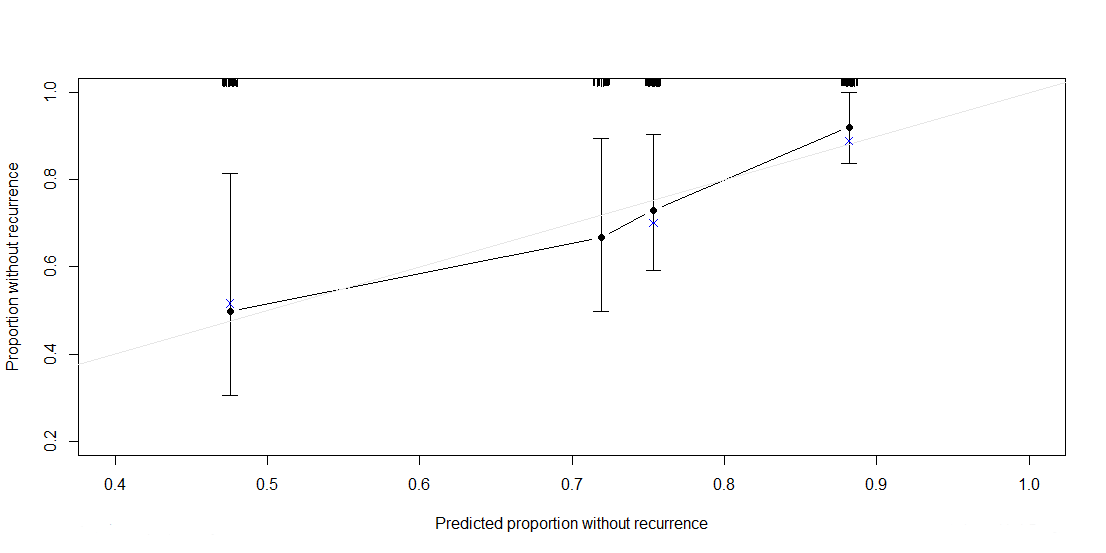

Supplement: Supplementary file 1 — Supplementary material 1 (TIFF 1813 kb) [file 10434_2016_5388_MOESM1_ESM.tiff]
